# Single-Cell Detection of *Erwinia amylovora* Using Bio-Functionalized SIS Sensor

**DOI:** 10.3390/s23177400

**Published:** 2023-08-24

**Authors:** Ui Jin Lee, Yunkwang Oh, Oh Seok Kwon, Jeong Mee Park, Hyun Mo Cho, Dong Hyung Kim, Moonil Kim

**Affiliations:** 1 Critical Diseases Diagnostics Convergence Research Center, Korea Research Institute of Bioscience and Biotechnology (KRIBB), 125 Gwahang-ro, Yuseong-gu, Daejeon 34141, Republic of Korea; waainie@eyebiokorea.com (U.J.L.); oyk0213@kribb.re.kr (Y.O.); 2 SKKU Advanced Institute of Nanotechnology (SAINT), Sungkyunkwan University, Suwon 16419, Republic of Korea; oskwon79@skku.edu; 3 Department of Nano Science and Technology, Sungkyunkwan University, Suwon 16419, Republic of Korea; 4 Department of Nano Engineering, Sungkyunkwan University, Suwon 16419, Republic of Korea; 5 Plant Systems Engineering Research Center, Korea Research Institute of Bioscience and Biotechnology (KRIBB), 125 Gwahang-ro, Yuseong-gu, Daejeon 34141, Republic of Korea; jmpark@kribb.re.kr; 6 Division of Advanced Instrumentation Institute, Korea Research Institute of Standards and Science (KRISS), 267 Gajeong-ro, Yuseong-gu, Daejeon 34113, Republic of Korea; hmcho@kriss.re.kr

**Keywords:** single-cell, *Erwinia amylovora*, fire blight, LptE, SIS, biosensor

## Abstract

Herein, we developed a bio-functionalized solution-immersed silicon (SIS) sensor at the single-cell level to identify *Erwinia amylovora* (*E. amylovora*), a highly infectious bacterial pathogen responsible for fire blight, which is notorious for its rapid spread and destructive impact on apple and pear orchards. This method allows for ultra-sensitive measurements without pre-amplification or labeling compared to conventional methods. To detect a single cell of *E. amylovora*, we used Lipopolysaccharide Transporter E (LptE), which is involved in the assembly of lipopolysaccharide (LPS) at the surface of the outer membrane of *E. amylovora*, as a capture agent. We confirmed that LptE interacts with *E. amylovora* via LPS through in-house ELISA analysis, then used it to construct the sensor chip by immobilizing the capture molecule on the sensor surface modified with 3′-Aminopropyl triethoxysilane (APTES) and glutaraldehyde (GA). The LptE-based SIS sensor exhibited the sensitive and specific detection of the target bacterial cell in real time. The dose–response curve shows a linearity (R^2^ > 0.992) with wide dynamic ranges from 1 to 10^7^ cells/mL for the target bacterial pathogen. The sensor showed the value change (d*Ψ*) of approximately 0.008° for growing overlayer thickness induced from a single-cell *E. amylovora*, while no change in the control bacterial cell (*Bacillus subtilis*) was observed, or negligible change, if any. Furthermore, the bacterial sensor demonstrated a potential for the continuous detection of *E. amylovora* through simple surface regeneration, enabling its reusability. Taken together, our system has the potential to be applied in fields where early symptoms are not observed and where single-cell or ultra-sensitive detection is required, such as plant bacterial pathogen detection, foodborne pathogen monitoring and analysis, and pathogenic microbial diagnosis.

## 1. Introduction

*E. amylovora* is a Gram-negative, rod-shaped bacterium belonging to the family *Enterobacteriaceae* [1,2]. Since its initial discovery in New York in 1780, fire blight, caused by an *E. amylovora* bacterium, has rapidly disseminated across various apple- and pear-producing regions, which is now present in more than 50 countries, including North America, New Zealand, and Europe [3]. The bacteria are a highly adaptable pathogen capable of colonizing diverse environments, including the leaves, stems, roots, fruit, and soil of its host plants [4,5]. Notably, *E. amylovora* exhibits remarkable resilience to environmental stressors such as freezing, drying, and heat [6,7]. The global prevalence, severity, and gravity of *E. amylovora*, a plant disease, has led to its categorization as a quarantine organism in numerous countries. The movement of plants and their derivatives from areas impacted by fire blight are strictly monitored by plant quarantine regulations. [2,8].

Studies have attempted to develop biosensors for the detection of *E. amylovora* [9]. Traditional methods such as culture and counting are the most common ways to confirm *E. amylovora* in standardized laboratories, but the time required to obtain results is prolonged, and the sensitivity to certain pathogens is constrained, with some pathogens being uncultivable [10]. Real-time or quantitative polymerase chain reaction (PCR) is mainly used for the detection of *E. amylovora*. Real-time PCR is a powerful tool for the diagnosis of plant diseases, but it has the disadvantage of requiring sample preparation for nucleic acid extraction and additional probe synthesis [11,12]. The use of digital PCR for the specific identification and quantification of viable *E. amylovora* cells has been demonstrated as a promising approach [13]. Nonetheless, it should be emphasized that the application of these methods is limited to laboratory settings and requires specialized training and expensive equipment. The use of a loop-mediated isothermal amplification (LAMP) method, one of the isothermal amplification PCR techniques, has been reported for the detection of *E. amylovora* [14,15]. However, this method can produce complex amplification products that are difficult to distinguish from background noise at low levels of infection. Besides molecular diagnostics, a lateral flow assay (LFA)-based rapid antigen detection test has been developed for the detection of fire blight owing to its easy handling, portability, and affordability [16,17]. Nonetheless, this technique has limitations in sensitivity and accuracy, thereby confining its use for primary screening. Bacterial pathogens need to be detected at an early stage, even if present in negligible numbers, to minimize the impact of bacterial infections from the detection viewpoint. This is especially critical for highly transmissible bacteria, where detecting them at the initial stage of infection, even before the manifestation of symptoms, is vital. Thus, the immediate requirement of a biosensor that possesses ultra-sensitive, real-time, and label-free features is necessary for the timely detection of fire blight. Through a literature review of previously reported sensing methods for *E. amylovora* detection, we confirmed that our sensor system possesses novelty in recognition element, rapid response time and ultra-high sensitivity. A comparison of various diagnostic methods for *E. amylovora* detection is described in Table 1.

A promising sensor platform based on silicon, called SIS sensor, has been developed that can replace the traditional gold film used in Surface Plasmon Resonance (SPR) sensors [20,21]. The traditional SPR sensors need an additional reference channel to eliminate the noise signal from refractive index changes of the buffer solution and the gold thin film induced by variations of surrounding environments such as temperature and flow conditions [22,23]. To address this issue, the SIS sensor was constructed using an in-house single-wavelength ellipsometer. At the non-reflecting condition for the *p*-polarized wave of the probing beam, that is the Brewster angle, the ellipsometric signal (*Ψ*) shows ultra-sensitive to overlayer thickness variations while the phase difference (Δ) almost remains unchanged. These factors reduce the noise signals induced from surrounding conditions, which allows for high signal-to-noise ratio on the affinity analysis of target antigens. In the current study, a bio-functionalized SIS sensor has been fabricated by immobilizing the LptE protein of *E. amylovora* as a capture agent on the silicon surface, enabling extremely sensitive detections with real-time monitoring manner for *E. amylovora* bacteria at the single-cell level.

## 2. Materials and Methods

### 2.1. Chemicals and Materials

3′-Aminopropyl triethoxysilane (APTES) and glutaraldehyde (GA) were obtained from Sigma Aldrich (Saint Louis, MO, USA). Silicon wafer (100) as the SIS sensor chip was purchased from MCL Electronics Materials (Luoyang, China), and 11.5 mm × 11.5 mm sensor chips were used for this work. Phosphate-buffered saline (PBS, pH 7.4) served as the binding and washing buffer.

### 2.2. Bacterial Strains and Culture Conditions

The *E. amylovora* strain used in this study was obtained from KRIBB. *E. amylovora* was cultured by inoculating a single colony onto LB agar (10 g/L tryptone, 10 g/L NaCl, 5 g/L yeast extract, 10 g/L agar) (Sigma Aldrich, Saint Louis, MO, USA) and subsequently transferring it to LB medium. The bacterial suspension was adjusted to an OD_600_ of 0.1 by dilution with LB medium and then incubated at 28 °C under shaking (220 rpm) conditions. Changes in absorbance at 600 nm were monitored until the bacteria entered the stationary phase after 24 h.

### 2.3. Gene Cloning and E. coli Transformation

BL21 (DE3) *E. coli* (Novagen, Madison, WI, USA) was used as host, and pET-21a (+) (Novagen, Madison, WI, USA) was used as vector for the expression of LptE. Restriction enzymes and DNA-modifying enzymes were purchased from Promega (Madison, WI, USA) and New England Biolabs (Ipswich, MA, USA). In order to clone the full-length gene encoding for LptE in *E. amylovora*, it was amplified with the forward primer (ACCATATGCGACATCCGATAGTCTCT) and the reverse primer (ACCTCGAGTCGGGTGTAGGAGTTGTC) via PCR. The resultant DNA fragment was then inserted into the pET-21a (+) plasmid using the NdeI/XhoI restriction enzyme cleavage sites, and the plasmid DNA was transformed into BL21 (DE3) cells for expression.

### 2.4. Protein Expression and Purification

*E. coli* cells were grown in a 10 mL LB starter culture overnight at 37 °C. One mL of the starter culture was inoculated in 100 mL of LB with ampicillin. Cells were grown at 37 °C with shaking until OD_600_ = 0.6. Cells were induced with 1 mM isopropyl-2-D-thiogalactopyranoside (IPTG) (GibcoBRL, Grand Island, NY, USA) and grown for 4 h. Cells were then harvested by centrifugation at 6000× *g* at 4 °C for 10 min. Harvested cells were resuspended in 50 mM Tris-HCl buffer (pH 8.0), and disrupted by sonication. The crude cell lysates were separated into total, soluble, and insoluble fractions, which were analyzed by 12% SDS-PAGE. In order to purify the recombinant proteins, 10 mL of the crude cell lysates were loaded onto an IDA-miniexcellose affinity column (Keyprogen, Daejeon, Republic of Korea). The recombinant proteins were subsequently eluted with 5 mL of 0.5 M imidazole in the same buffer (50 mM sodium phosphate, pH 8.0). The protein concentration was determined by Nano drop (Thermo scientific, Waltham, MA, USA). Finally, recombinant LptE protein was concentrated to 100 mg/mL, and stored at −80 °C for further experiments.

### 2.5. LPS Extraction

LPS was extracted using an LPS extraction kit (Intron Biotechnology, Seongnam-Si, Republic of Korea) with some modifications. Briefly, cells were harvested and lysed in lysis buffer (50 mg of cells/mL of lysis buffer) and then subjected to a vigorous vortex to dissolve cell clumps. After the addition of chloroform, the sample was centrifuged for 45 min at 4 °C. The upper aqueous layer was collected, and 2 volumes of purification buffer were added to 1 volume of aqueous layer. The mixture was then incubated at −20 °C for 2 h, before centrifugation at 20,000× *g* for 10 min at 4 °C. The resulting LPS samples were purified to remove any remaining particulates and stored in sterile vials at 4 °C.

### 2.6. In-House ELISA

Microplates were coated overnight at 4 °C with 75 uL/well of 1 ug/mL poly-L-lysine (Sigma Aldrich, Saint Louis, MO, USA) diluted in 0.05 M bicarbonate ELISA coating buffer. The poly-L-lysine-modified microplate was incubated with 10^4^ cells/well of *E. amylovora* in PBS for 20 min at room temperature. Serial dilutions from LptE proteins in PBS, covering a wide range of concentrations from 0.1 to 15 ug/mL, were prepared and added. Following an hour of incubation at 37 °C, three washes with PBS were performed followed by incubation with primary antibody (His-tag antibody) for 1 h at 37 °C. Then, three washes with PBS were performed followed by the addition of HRP-conjugated secondary antibody (anti-mouse IgG). After the final three washes with PBS, the plate was developed with 100 uL/well of peroxidase substrate for 5 min, and 50 uL/well of H_2_SO_4_ was added as a stop solution. The optical density was measured at 450 nm in an ELISA plate reader.

### 2.7. Surface Modification

In order to immobilize LptE onto a silicon surface, a series of surface modifications were performed. Firstly, the silicon chips were washed using ethanol solution to remove organic contaminants and particles, and then the sensor surface was modified with a 2 vol% APTES in ethanol for 12 h. The self-assembled monolayer of APTES was treated with a 2 vol% solution of GA in PBS for 2 h. Following this, the APTES-GA-modified surface was utilized for bio-functionalization with LptE as a capturing agent.

### 2.8. Atomic Force Microscopy (AFM) Analysis

For the AFM analysis, LptE (100 mg/mL in a PBS buffer, pH 7.4) was dried on top of the APTES-GA-modified SiO_2_ surfaces, and scanned with a multimode Nanoscope V controller. AFM images were obtained in the tapping mode under ambient conditions using the Igor Pro 6.36 program. The height and the roughness were determined from horizontal line scans (n = 3 for each crater, 3 craters/sample).

### 2.9. SIS Measurement

The LptE-modified SIS sensor system used a p-type silicon wafer (100) cut to 11.5 mm × 11.5 mm as a sensor chip applied to the sensor cell tilted 2° to the prism surface. The PBS was allowed to flow for about 2–5 min until the sensor signal stabilized. The incident angle of the probing light was automatically adjusted at the Brewster angle with respect to the sensor chip using the goniometer (Huber Corp., Edison, NJ, USA) mount on the SIS system which can provide a high accuracy of 0.001°. One-hundred ug/mL of LptE protein (PBS, pH 7.4) was prepared and utilized in this study, as optimized in our previous study, and the flow rate was 30 uL/min. The sensor layer was washed thoroughly with PBS solution to remove unbound or weakly bound LptE proteins on the surface. The target samples were injected over the bio-functionalized SIS surface, and unbound bacterial pathogens were removed using PBS for 5 min. The reactive values for target bacteria were subtracted by the non-specific signals and were analyzed by averaging the measured signals for 3 min at the washing step.

## 3. Results and Discussion

### 3.1. Principle of SIS Sensor System

The SIS sensor is a polarizer–sample–analyzer (PSA) system that measures the characteristics of a target analyte by analyzing changes in the polarization state of light that is reflected from a sample with a specific polarization state. The SIS sensor operates sensitively to ellipsometric parameters (*Ψ*, Δ); *Ψ* and Δ are the amplitude ratio and phase difference between *p*-polarized and s-polarized lights as electric fields aligned in parallel and perpendicular, respectively, to the plane of incidence. These parameters are defined by the complex reflectance ratio ρ as

ρ = rp/rs = tan(*Ψ*)eiΔ (1)

where rp and rs are the complex reflection coefficients of *p*-polarized and s-polarized light; *Ψ* = tan^−1^ (|ρ|); and Δ = δp − δs. The SIS sensor measures at a non-reflecting condition for the *p*-polarized wave where *Ψ* shows sensitivity for the thickness change while Δ is almost constant; therefore, *Ψ* is used as a sensing parameter which detects the variation of surface thickness due to the binding events on the sensor surface.

**Figure 1:**
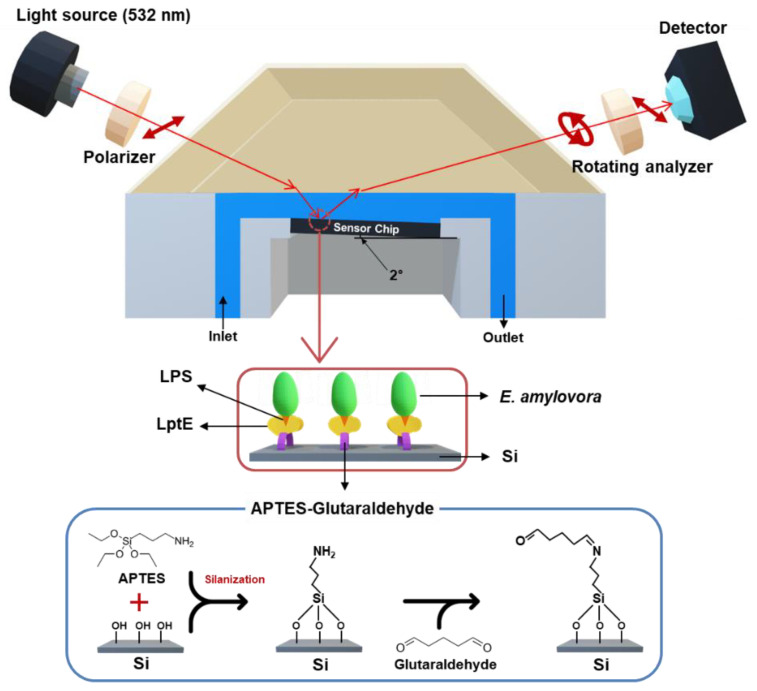
A schematic illustration depicting the LptE-modified SIS sensor designed for the purpose of detecting *E. amylovora*. The SIS system includes several components such as a light source, a fixed polarizer, an SIS sensor assembly, a rotation analyzer, and a detector. At the Brewster angle, the incident light of 532 nm wavelength is directed towards the sensor via a prism that induces the incident and reflected beam path. For the immobilization of LptE onto the silicon surface, the SIS surface was treated with APTES (2%) and GA (2%) sequentially. The APTES-GA-modified surface was subsequently bio-functionalized with LptE as a capture agent.

Figure 1 shows a schematic diagram of the bio-functionalized SIS sensor which consists of a probing beam, fixed polarizer, SIS assembly, rotating analyzer, and detector. In the SIS sensor, a single-wavelength laser with a wavelength of 532 nm was used for high-sensitivity measurement. Light emitted from a light source changes to a specific polarization state after passing through a polarizer. When this polarized light is reflected from the SIS surface, its polarization characteristics are altered, and the reflected light passes through a rotating analyzer before being measured by a detector. For the real-time analysis of bacterial adhesion characteristics, a fluidics system consists of an auto-isolation valve, flow channel, and syringe pump. The flow channel was provided in the SIS assembly which is composed of the silicon chip, sensor cell, and optical prism. The sensor cell supports the flow channels over the sensor chip, applying a special structure that induces a 2° tilt of the chip with respect to the prism surface. The 2° tilted design can remove the noise light reflected from the prism surface, resulting in an increase in the signal-to-noise ratio (SNR). The chip was functionalized to be covalently bound with a bio-receptor, which leads to better stability of the sensing signal than physical adsorption means. The ellipsometric parameters calculated as a result of the interaction between the analyzed substance and the sensor surface can evaluate the characteristics of the sample or the presence of target analytes in the sample.

### 3.2. Growth Curve of E. amylovora

**Figure 2:**
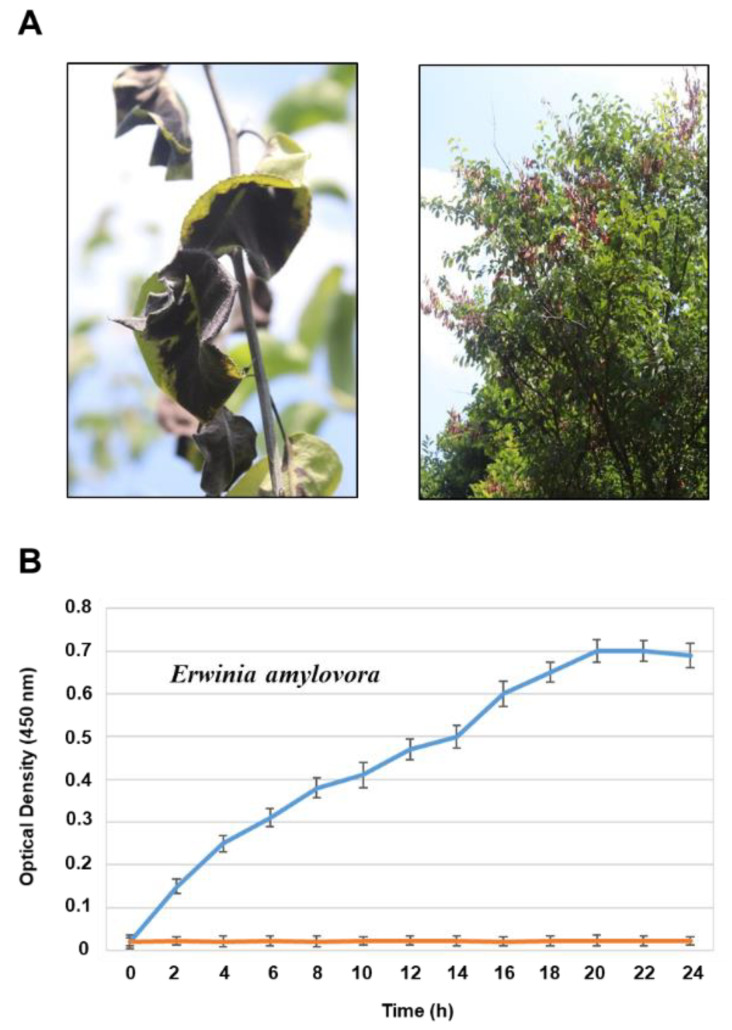
(**A**) The scorched appearance of fire blight on infected pear trees (photo by Dr. M. Kim). (**B**) Growth curve of *E. amylovora*, the causative agent of fire blight. The growth curve of *E. amylovora* (blue graph) was determined by monitoring changes in its optical density (OD_600_) under shaking culture conditions (220 rpm) at 28 °C for 24 h. The graph in orange represents LB media only. Optical density was measured every 2 h. Error bars indicate the standard deviation. The experiment was performed with three biological replicates.

Figure 2A shows the burnt appearance that fire blight causes on pear trees. When a pear tree is infected with fire blight, the affected leaves and shoots typically exhibit a discoloration that ranges from brown to black. In cases where there are several infected shoots present on a single tree, the overall appearance of the tree may resemble that of being scorched by fire, hence giving the name “fire blight”. The initial stage in conducting a diagnostic study on fire blight is to obtain the causative agent of the disease, which is *E. amylovora*. The strain of bacteria obtained from KRIBB was cultured and utilized for further detection tests. To quantify the bacterial cell numbers of *E. amylovora*, we observed its growth status by measuring the optical density (OD_600_) for 24 h. The properties of bacterial growth were recorded under shaking culture conditions (180 rpm). As shown in Figure 2B, the growth curve of *E. amylovora* showed two phases, the log or exponential phase (0–20 h) and stationary phase (20–24 h), which is consistent with a previously reported bacterial population growth curve [22]. Based on the bacterial growth curve, the population of *E. amylovora* was calculated, as the number of *E. amylovora* was defined as follows: OD_600_ 0.1 = 1 × 10^8^ cells/mL. The *E. amylovora* yielded a cell count of approximately 7 × 10^8^ cells/mL at 20 h after incubation, and remained at that level.

### 3.3. Structure of LptE

**Figure 3:**
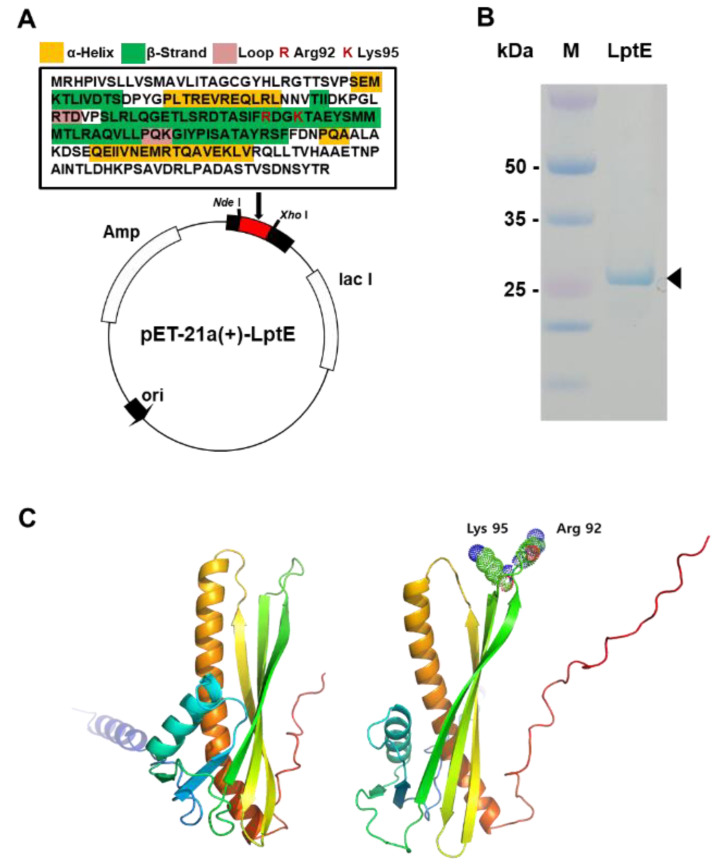
(**A**) Amino acid sequence characteristics of LptE and construction of recombinant plasmid pET-21a(+)-LptE for expression of LptE. Yellow boxes (α-Helices) and green boxes (β-Strands) and pink boxes (Loops) represent secondary structural components. Red-coded R (Arg 92) and K (Lys 95) indicate positively charged amino acids predicted to be a necessary requirement for the interaction of LPS. (**B**) SDS-PAGE analysis of the recombinant LptE. After IPTG induction, purified recombinant protein was analyzed via 10% SDS PAGE. The arrowhead indicates the expressed 25-kDa LptE. (**C**) Modeling of LPS-free LptE structure (left) and LptE in binding complex with LPS (right). LPS interacts with positively charged residues (Arg 92 and Lys 95) on the outer surface of the β-strands of LptE.

The map of recombinant pET-21a (+)-LptE expression vector is presented in Figure 3A. The secondary structural components, namely α-helices denoted by yellow boxes, β-strands denoted by green boxes, and loops denoted by pink boxes, are depicted in the figure. Additionally, positively charged amino acids, R (Arg 92) and K (Lys 95), are highlighted in red and predicted to be crucial for the interaction with LPS. After IPTG induction, there was an obvious band around the molecular weight of 25 kDa, which is consistent with the expected molecular weight of recombinant LptE protein (Figure 3B). Through protein structure-based modeling via PyMOL, it has been verified that two positively charged residues (Arg 92 and Lys 95) in the outer loop region of LptE provide electrical interaction with LPS (Figure 3C). The amino acid residues of the LPS binding site are exposed on the surface, facilitating the binding of LPS to the interacting protein without causing steric clashes [24]. Structural studies have shown that the lipid A portion of LPS, which is the most invariant region, selectively interacts with positively charged residues on the outer surface of the beta-sheet of the LPS-binding proteins [25,26]. This can be attributed to the suitability of positively charged residues to bind with LPS, as LPS interacts with negatively charged sugars, such as phosphates and negative partial charges. In this study, we utilized LptE, which has previously been shown to interact with LPS from *E. amylovora*, as a capture agent for the first time. The significant benefit of using LptE as a capture agent lies in its capability to recognize bacteria directly from samples without the need for prior amplification. In addition, to enhance the detection performance, it is possible to utilize modifications of LptE. For example, LptE can be engineered through directed molecular evolution screening to increase its affinity for its cognate LPS. Nevertheless, the detection of *E. amylovora* using LptE requires comprehensive studies, including the optimization of the protocol and the assessment of different strains and plant samples, to evaluate the practicality and reliability of this diagnostic method.

### 3.4. In-House ELISA for Verifying LptE Interaction with E. amylovora

**Figure 4:**
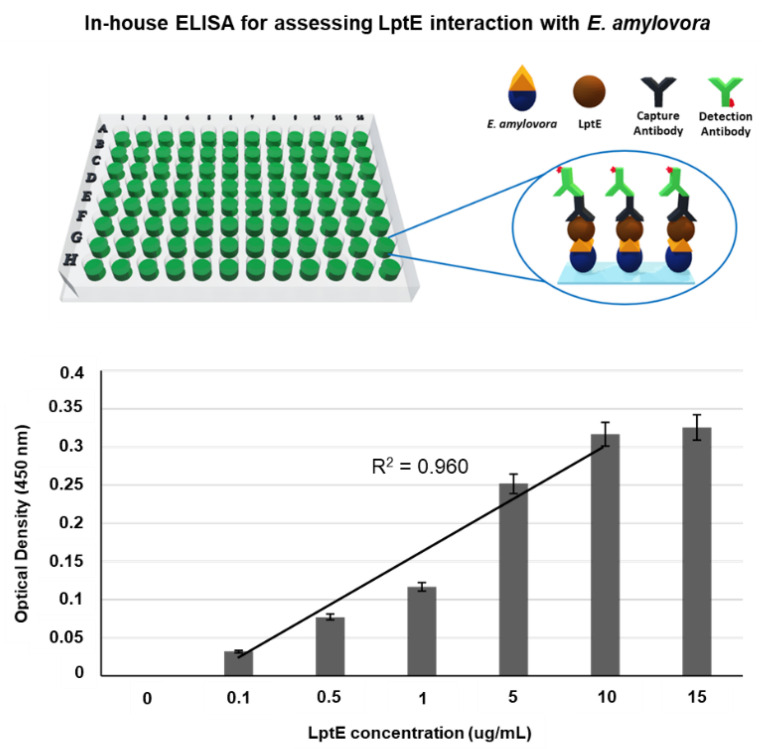
In-house ELISA for assessing LptE interaction with *E. amylovora*. Plate was coated with 10^4^ cells/well of *E. amylovora*, and LptE proteins were serially diluted (as indicated) and added. The binding complex of *E. amylovora* and LptE was analyzed by adding the primary antibody (His-tag antibody) and HRP-conjugated secondary antibody (anti-mouse IgG), sequentially. The optical density was measured at 450 nm in an ELISA plate reader.

To validate the interaction between LptE and *E. amylovora*, an in-house ELISA analysis was performed. For this purpose, a whole-bacterial-cell ELISA using bacterial cells as antigens was developed. In particular, poly-L-lysine was introduced onto the surface of microplate wells to immobilize bacterial cells, based on the notion that the cationic polypeptide interacts with the negatively charged cell surface via ionic adsorption. As shown in Figure 4, the observed changes in optical density at 450 nm were found to be proportional to the concentration of the tested LptE. The binding affinity between LptE and *E. amylovora* was calculated as Kd = 1.44 ug/mL (R^2^ = 0.960), showing the affinity between LptE and the target bacterial cells. LptE can interact with bacterial cells via LPS recognition. LPS-based ELISA analysis showed that changes in signal intensity were proportional in response to the concentration of the tested LptE ranging from 0.1 to 10 ug/mL (data not shown), indicating the association of LptE with LPS, the major component of the outer membrane of Gram-negative bacteria. The results imply that LPS-binding protein can be useful for the diagnosis and monitoring of Gram-negative bacteria.

### 3.5. Immobilization of LptE

**Figure 5:**
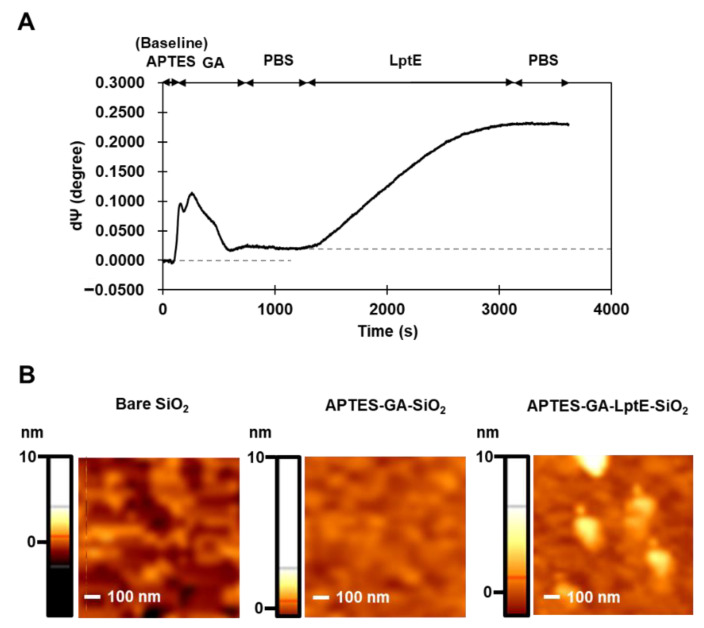
Immobilization of LptE onto the SIS sensor chip. Measurement of the thickness of APTES-GA-LptE layer using SIS ellipsometry (**A**). AFM images of APTES-GA and APTES-GA-LptE coating on the SIS silica surface (**B**).

The immobilization of LptE as the capture molecule was achieved on silicon surfaces modified with APTES-GA. For the implementation of the sensor chip to *E. amylovora* bacterial cell detection, LptE was applied to the sensing area of SIS sensor for 3 min, followed by a subsequent 1 min PBS buffer wash to eliminate unbound molecules. As shown in Figure 5A, the attachment of GA to the APTES-modified SiO_2_ surface served as the baseline, resulting in a marginal alteration of *Ψ* signal due to the thickness variation of the chip, approximately 0.0221. Upon immobilization of LptE onto the APTES-GA-modified silica surface, the *Ψ* value exhibited a substantial increase to approximately 0.2307, and the binding level remained consistent during the washing step. The experimentally obtained *Ψ* value of 0.2307 can be converted to an estimated surface thickness of approximately 1.0185 nm. Given that LptE protein forms a roll-like structure, this value aligns with the estimated molecular size of LptE (25 kDa in molecular weight, 5 nm in length, 1 nm in thickness).

The SIS surface modified with LptE was imaged through AFM to investigate the roughness of the LptE layer. Figure 5B shows AFM 3D images of bare SiO_2_, APTES-GA and APTES-GA-LptE deposited on the SIS silica surface. Software for AFM (Dimension 3100, Veeco Inc.) was used to calculate the average surface roughness (root mean square roughness, Rq) of each AFM image. Rq values were determined as follows: 0.21 nm for bare SiO_2_, 0.61 nm for APTES-GA-SiO_2_, and 2.69 nm for APTES-GA-LptE-SiO_2_, demonstrating the binding of the protein of interest on the SIS surface.

### 3.6. Single-Cell Detection of E. amylovora

**Figure 6:**
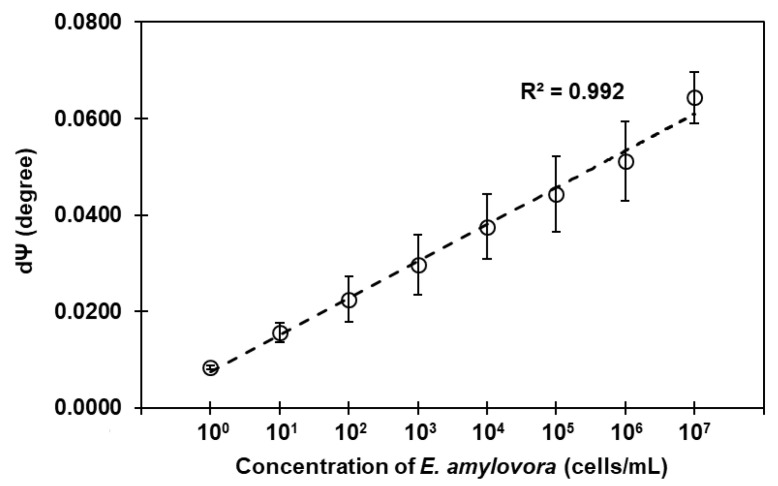
Dose–response curve to *E. amylovora*. The d*Ψ* values at each dose were plotted against the *E. amylovora* concentrations between 1 and 10^7^ cells/mL, linearized by log transformations. The SIS signal intensities were expressed in d*Ψ*.

The most important feature of ellipsometry as a sensing technique of the SIS sensor is the simultaneous measurement of refractive index and thickness, which can provide accurate detection, even for very low-molecular-weight or low-concentration biomolecules on the sensor surface. Specifically, the SIS sensor operates by directing incident light through a buffer solution, a bio-layer, and a substrate layer (SiO_2_). In this configuration, the buffer solution serves as the incident medium, whose role is to provide a constant temperature and humidity environment; errors due to refractive index changes are extremely eliminated at the Brewster angle, resulting in a sensitivity more than 10 times higher than conventional ellipsometric sensors [21,22]. Using the SIS sensor system, we analyzed the interaction between *E. amylovora* and the LptE-functionalized SIS surface. Figure 6 shows the dose–response curve plotted against the concentrations of each sample by a logarithmic scale, which displays a linearity (R^2^ > 0.992) for wide dynamic range between 10^0^ and 10^7^ cells/mL. The coefficient of variation for triplicate measurements was within 21.0% at the bacterial detection point.

**Figure 7:**
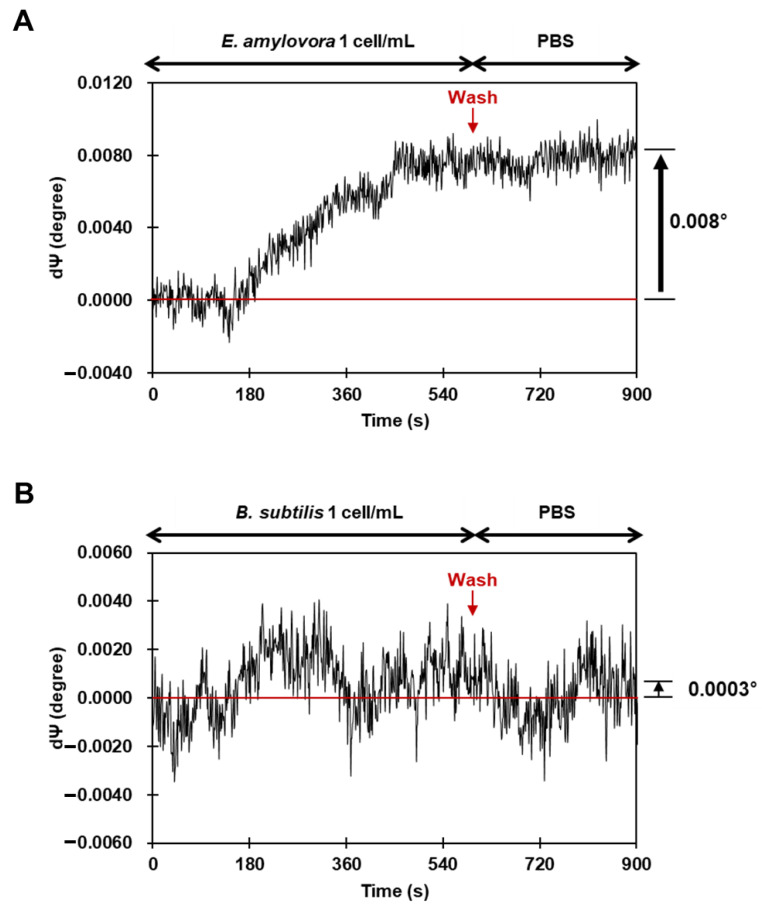
Single-cell detection of *E. amylovora*. The plot of d*Ψ* versus time after injection of (**A**) *E. amylovora* and (**B**) other bacterial species (*B. subtilis*) at a single-cell level on the LptE-modified SIS sensor. The SIS signal intensities were expressed in d*Ψ*.

At the Brewster angle, in our case at 72.18°, the change in *Ψ*, d*Ψ*, varies sensitively with thickness change due to the interaction of analytes with the sensor surface. Based on the numerically simulated behavior of the SIS sensor [20], the d*Ψ* value measured by SIS can be theoretically converted into bio-layer thickness (dT) using the following conversion equation:

dT = 1000 × d*Ψ*/0.2265 (pm) (2)

where 1/0.2265 is a thickness translation factor, depending on the wavelength of probing light (532 nm) and 1000 is the scaling factor. It means one nm change in thickness will alter the value of *Ψ* by 0.2265 within the optimized conditions. According to the data shown in Figure 7A, it can be estimated that the 532 nm laser gives d*Ψ* = 0.008° for a 35.32 pm thickness change. If we consider the average size of *E. amylovora* to be around 5 µm in diameter, the volume of the bacterial cell can be calculated to be approximately 65 femtoliters. Assuming that the volume corresponding to a single cell is passivated on the SIS sensor cell (1.5 mm × 6.0 mm) and interacted with the inclined beam with a major axis of <6.54 mm and a minor axis of <2.0 mm, the thickness is calculated to be approximately 7.22 pm. If target substances are evenly distributed on the surface, an overall change in refractive index across the surface is measured. However, in the case of single-cell adhesion to the surface, where a single and large analyte is distributed at a specific point, the change in refractive index at that point will be much greater. Therefore, it can be speculated that the reason why the experimental data differ from the calculated value is that the refractive index change can vary depending on the adsorption state and location of the analytes. In order to evaluate the specificity of the LptE-SIS sensor, we performed SIS measurement in response to *B. subtilis*, a Gram-positive bacterium that does not contain LPS. As shown in Figure 7B, the d*Ψ* value of *B. subtilis* showed a negligible increase of approximately *Ψ* = 0.0003, which is only about 3.7% of the increment observed in *E. amylovora*. Although specificity analysis for various bacterial strains was not performed in the current study, this result demonstrates that at least the ellipsometric angle of the LptE-modified SIS sensor is not affected by LPS-free *B. subtilis*.

**Figure 8:**
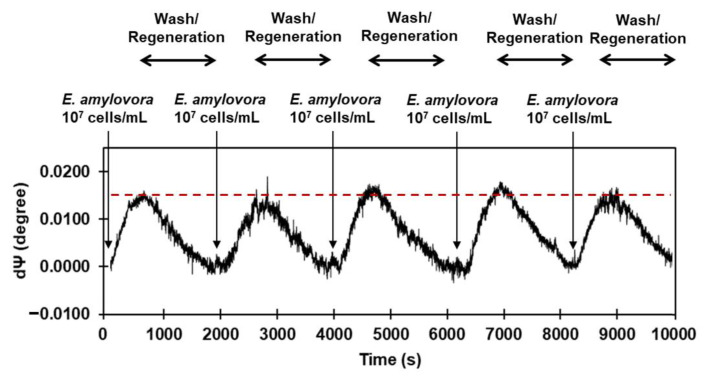
Reusability of LptE-modified SIS sensor for *E. amylovora* detection. Sequential steps of bacterial injection (10^7^ cells/mL), wash at low flow rate (30 uL/min) and regenerate at high flow rate (100 uL/min) at least 5 times over 10,000 s, being continuously monitored on the same surface without additional surface modification.

Another important aspect of biosensors is the sensor’s surface regeneration. Biosensors are often designed for disposable measurements, and to reuse a biosensor for bacterial detection, a complex surface/interface architecture needs to be employed followed by regeneration. Regeneration typically involves the removal of bacterial cells and underlying linker layers and capture agents on the sensor surface, along with surface modifications to facilitate the re-binding of bacteria. This process is often complex and time-consuming. Therefore, we attempted to investigate the surface regeneration and reusability potential of the SIS sensor modified with LptE utilizing a physical regeneration method involving an enhanced flow rate, which can avoid hindrance to the binding capacity of receptor proteins due to the use of a regeneration buffer containing glycine [27]. This approach is based on the notion that the robust cross-linking of LptE-functionalized SIS surfaces through covalent bonding with GA enables enhanced flow resistance, whereas the significantly larger *E. amylovora* (5 um in diameter, 65 fL in volume), non-covalently linked with LptE, is unable to endure such conditions and readily dissociates from the surface. As shown in Figure 8, the sequential injection of 10^7^ cells/mL *E. amylovora*, washed at a low flow rate (30 uL/min) and regenerated at a high flow rate (100 uL/min), was repeated five times over 10,000 s for continuous signal measurement of SIS in response to *E. amylovora*. The observed magnitude of SIS signal change in the first reusability test was 0.0149, and consistent signal peaks were maintained without signal reduction until the fifth reusability test. From the results obtained from the reusability tests, it was confirmed that the sensor could be reused for the detection of *E. amylovora* through surface regeneration modified with LptE, which is advantageous in terms of the cost-effectiveness aspect of the sensor.

Recently, Ivanov et al. reported a comparison of the detection limit for *E. amylovora* based on different isothermal amplification methods [9]. In that study, the detection limit was 10^4^ cells/mL for LAMP, 10^3^ cells/mL for LAMP-CRISPR/Cas, and 10^2^ cells/mL for recombinase polymerase amplification (RPA) and RPA-CRISPR/Cas. To our knowledge, the single-cell detection of *E. amylovora* has not been reported before. It is assumed that the excellent outcome obtained from this work is due to the integration of an ultra-sensitive SIS sensor and a bio-receptor capable of recognizing LPS from bacteria. In general, bio-functionalized sensors exhibit higher sensitivity with smaller sizes of bio-receptors. This is because smaller bio-receptors can detect subtle changes at the molecular level and respond to smaller energy changes. This study employed a relatively large protein, LptE, with a molecular weight of around 25 kDa, as a capture agent for bacteria. If domain function analysis can identify the minimal size necessary for LPS binding within the LPS-binding domain (LBD), the resulting bio-receptor could potentially exhibit a faster binding rate and higher sensitivity, thereby serving as an ideal capture agent. Therefore, the next study will focus on bacterial detection using a pre-functionalized SIS sensor with a minimal LBD.

## 4. Conclusions

To prevent the spread of fire blight and minimize its damage, there has been an increasing demand for early detection of the causal agent, *E. amylovora*. In this study, an ultra-sensitive bio-receptor-based SIS sensor capable of detecting *E. amylovora* at the single-cell level has been developed. LptE, a lipopolysaccharide-binding protein capable of recognizing the glycolipid on the bacterial surface, was used as the capture agent to capture *E. amylovora*. The bacteria-detecting LptE was found to interact with *E. amylovora* through in-house ELISA testing, and this was confirmed to be the result of direct binding between LptE and its cognate LPS. The bio-functionalized SIS sensor exhibited a wide dynamic range with linearity (R^2^ > 0.992) spanning from 10^0^ cells/mL to 10^7^ cells/mL, highlighting its capability to detect *E. amylovora* at the single-cell level, with no observed signal change for the positive Gram bacterium (*B. subtilis*). Furthermore, the LptE-modified SIS sensor demonstrated effective reusability for the continuous detection of *E. amylovora*. The great advantage of using the LptE-modified SIS is that genetically engineered receptors with improved affinity and specificity for the target material can be easily produced. Therefore, a superior bio-functionalized SIS can be introduced by designing a more effective bio-capture agent for the target bacteria. In field tests where immediate bacterial detection without the need for culturing pathogenic bacteria is imperative, there exists a critical demand for highly sensitive sensors. In this context, the current study, which has achieved the single-cell detection limit for *E. amylovora*, sheds light on the potential applicability of our system in the field of primary field screening requiring high-sensitivity detection and large-scale sample monitoring.

## Figures and Tables

**Table 1:** Comparison of various diagnostic methods for *E. amylovora* detection.

Sensor Type	Recognition Element	Response Time	Limit of Detection	Ref.
Conventional PCR	EtBr	180 min	10^3^ cells/mL	[12]
Real-time PCR	TaqMan	60 min	10^2^ cells/mL	[12]
LAMP	FAM	15 min	1.2 × 10^4^ CFU/mL	[15]
LFA	pAb	10 min	4 × 10^5^ CFU/mL	[16]
ddPCR	PicoGreen	80 min	5 × 10^3^ CFU/mL	[18]
Fluorescent probe	B-1	10 s	10^2^ CFU/mL	[19]
SIS	LptE	15 min	10^0^ cells/mL	This study

## Data Availability

The data presented in this study are available on request from the corresponding author (M.K. or D.H.K.).
